# Viscoelastic coarsening of quasi-2D foam

**DOI:** 10.1038/s41467-023-36763-y

**Published:** 2023-02-28

**Authors:** Chiara Guidolin, Jonatan Mac Intyre, Emmanuelle Rio, Antti Puisto, Anniina Salonen

**Affiliations:** 1 Laboratoire de Physique des Solides, Université Paris-Saclay, CNRS, Orsay, 91405 France; 2 Department of Applied Physics, Aalto University, Espoo, 02150 Finland

**Keywords:** Fluid dynamics, Soft materials

## Abstract

Foams are unstable jammed materials. They evolve over timescales comparable to their “time of use", which makes the study of their destabilisation mechanisms crucial for applications. In practice, many foams are made from viscoelastic fluids, which are observed to prolong their lifetimes. Despite their importance, we lack understanding of the coarsening mechanism in such systems. We probe the effect of continuous phase viscoelasticity on foam coarsening with foamed emulsions. We show that bubble size evolution is strongly slowed down and foam structure hugely impacted. The main mechanisms responsible are the absence of continuous phase redistribution and a non-trivial link between foam structure and mechanical properties. These combine to give spatially heterogeneous coarsening. Beyond their importance in the design of foamy materials, the results give a macroscopic vision of phase separation in a viscoelastic medium.

## Abstract (web summary)

Understanding how foams destabilize is key for developing applications. Experiments with foamed oil-in-water emulsions now show that bubble size evolution can be controlled by varying the continuous phase elastic modulus, exploiting the interplay between a foam’s structure and mechanical properties.

## Introduction

Many soft matter materials, including liquids, polymers, and proteins, evolve through surface tension-driven phase separation phenomena. During this process, the growth of domains can occur via material transfer through the continuous phase. A particular example is foams, which coarsen as gas diffuses between bubbles due to differences in Laplace pressure. The rate of coarsening is set by foam topology through the organisation of the neighbouring bubbles^1,2^. The growth law is theoretically predicted and experimentally verified in two limiting cases of very dry or very wet foams. In both cases the foam reaches a self-similar scaling state with invariant statistical distributions^3^, which leads to a power-law growth of the mean bubble size 〈*R*〉. In dry foams gas diffusion occurs exclusively through the thin films separating the bubbles and 〈*R*(*t*)〉 ∝ *t*^1/2^, while in dilute bubble dispersions, the gas transfer through the bulk phase is captured within a mean-field description and 〈*R*(*t*)〉 ∝ *t*^1/3^^4^.

If gas diffusion is no longer the rate-limiting step in the coarsening process, the evolution could be different. In wet foams made from viscoelastic fluids, lamellar^5^ or smectic^6^, exponents below 1/3 have been measured. Bubbles in a viscoelastic continuous phase stop coarsening if the surrounding material is sufficiently stiff to oppose the growth (or shrinking) of the bubbles. The conditions for arrest have been predicted for both single bubbles and foams^7,8^. Bey et al. worked with monodisperse bubbles to show that, indeed, if the elastic energy of the medium is higher than the surface energy coarsening is halted^9^. Lesov et al. and Feneuil et al. measured an arrest once the continuous phase yield stress was higher than Laplace pressure^10,11^.

The elastocapillary number Ca_el_^12^ compares surface tension effects to bulk elasticity. In the case of capillary inclusions (liquid drops or gas bubbles) in an elastic matrix, Ca_el_ is defined as the ratio of the bulk elastic modulus *G*_0_ to Laplace pressure, thus ${\rm {C{a}}}_{{{{{{{{\rm{el}}}}}}}}}=\frac{{G}_{0}}{\gamma /R}$, where *γ* is the surface tension and *R* the bubble (drop) size. Depending on Ca_el_, the inclusions can either soften or stiffen the material^13,14^. In a foam, the bubbles cannot be considered spherical inclusions, as they are deformed with thin films between them. However, Ca_el_ has been shown to be the relevant parameter to describe foam elasticity due to the additivity of the bubble and continuous phase contributions^15,16^. Beyond the elastic regime, it is the yield stress of the continuous phase, which should be compared to Laplace pressure^17^.

In practice, we encounter foamy materials in a broad range of Ca_el_. Between the limits of capillary-controlled foams (e.g. soap froths) and solid foams, lies a number of materials with intermediate Ca_el_. These are foams made from soft solids (creams, pastes, gels) or precursors of solid foams. In these systems, Ca_el_ evolves in time, due to an increase of the bubble size (coarsening, coalescence) or solidification of the continuous phase, both of which lead to an increase of Ca_el_. Understanding bubble size evolution in such systems is crucial as it is a key control parameter of the material properties^18^. We thus explore the evolution of the bubble size and foam structure in a foam made from a model viscoelastic fluid (an emulsion), in which we vary the initial elastocapillary number from 1 to 8. In this range we expect the continuous phase to impact the coarsening process without arresting it.

## Results and discussion

**Fig. 1:**
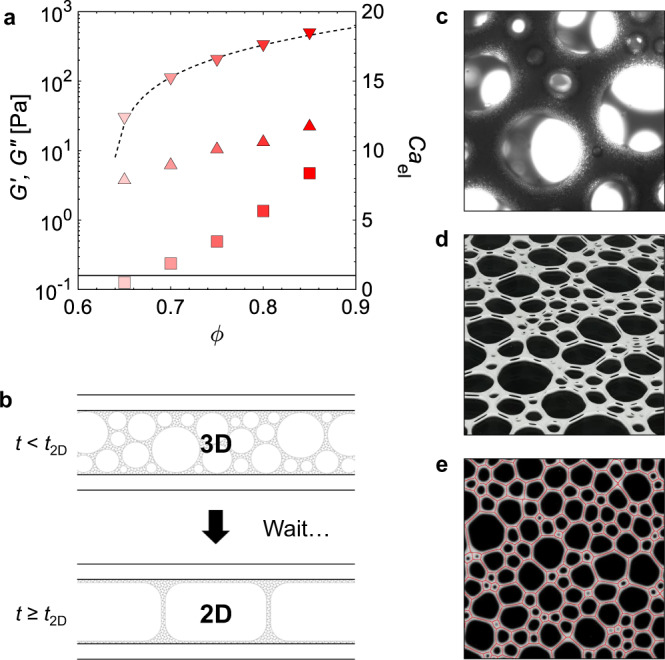
Foamed emulsion setup. **a** Storage (down triangles) and loss (up triangles) moduli of the emulsions used as the foam continuous phases as a function of oil volume fraction *ϕ*. The dashed line shows the expected scaling of ${G}^{{\prime} } \, \approx \, \phi (\phi -{\phi }^{*})$ with ϕ^*^ = 0.635 from ref. ^19^, which describes the data well. In the following, we use *G*_0_ for ${G}^{{\prime} }$ for the emulsion elastic modulus. The squares are Ca_el_ calculated using 〈*R*〉 = 0.5 mm and a surface tension of 30 mN/m and the solid line guides to Ca_el_ = 1. **b** Schematic drawing of the sample generation from 3D foam to the quasi-2D foam. The gap between the plates is 1 mm. **c** Photograph of foam after generation seen under the microscope. The edge size is 830 μm. **d** Perspective view of a quasi-2D foam. **e** Top view of foam with its skeleton overlaid in red. The edge size is 30 mm.

In concentrated emulsions, the elastic and viscous moduli can be easily varied by changing the volume fraction *ϕ* of the dispersed phase^19^. This is why we use oil-in-water emulsions, with an average drop size of 2 μm and *ϕ* between 0.65 and 0.85, which allows us to vary the continuous phase elastic modulus from 31 to 506 Pa (Fig. 1a). All the studied emulsions are predominantly elastic, the loss modulus always being <10% of the storage modulus. We use sodium dodecyl sulfate (SDS) at 30 g/L as the surfactant to stabilise both the oil/water and the gas/water interfaces. The choice of such a high SDS concentration ensures that all the interfaces are well covered and the emulsion drop size distributions are unchanged within the foams, over their entire lifetime.

We choose to work with quasi-2D foams, which consist of a single layer of bubbles sandwiched between two glass plates (Fig. 1d). Such systems have been widely used by the foam community to explore many aspects of foam stability^20,21^ and rheology^22^. The coarsening laws are the same as in 3D foams, but the specific configuration allows the tracking of individual bubbles for a fine analysis of the foam structure^23^. Instead of creating the foam directly between the plates, we first make a 3D foamed emulsion with small polydisperse bubbles and a liquid (emulsion) fraction *ϵ* ≈ 11% using a mixer to incorporate air in the emulsion (Fig. 1c). Then we fill and close the cell to leave the bubbles to grow. Once the average bubble diameter surpasses the gap size, so once 2〈*R*〉 ≳ *d*_gap_, the foam has become 2D and we define this time as *t*_2D_ (Fig. 1b). This allows us to start with quasi-2D foams with small, polydisperse bubbles (Fig. 1d). The images are skeletonised to measure the bubble size (Fig. 1e). We use 〈*R*〉 = 0.5 mm to calculate the initial Ca_el_, which for the different samples increases from 0.5 to 8 with increasing *ϕ* (Fig. 1a).

**Fig. 2:**
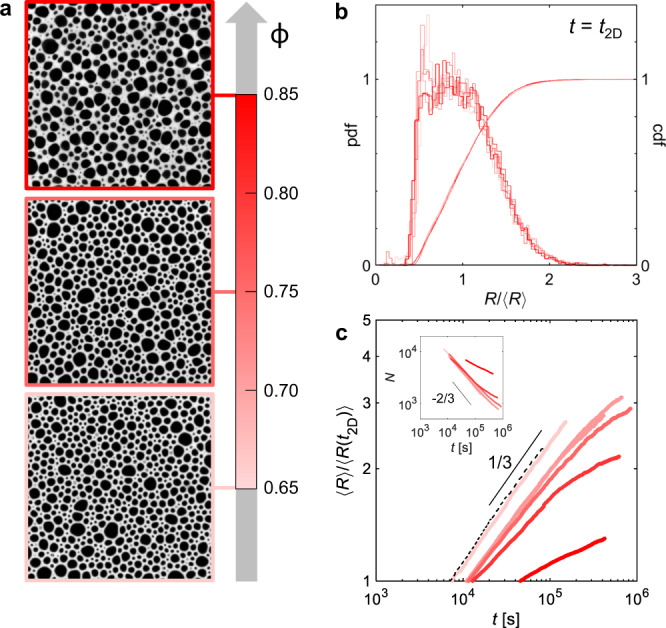
Initial foams and size evolution. **a** Photographs of foams made from the different emulsions at *t* = *t*_2D_. From top to bottom: *ϕ* = 0.85, 0.75, 0.65. The edge of each frame is 40 mm. **b** Bubble size distributions at *t* = *t*_2D_ for the five different foams studied, on the left axis the probability distribution function and on the right axis the cumulative distribution function. The initial distributions of the foams are almost identical, and the cumulative distributions overlap almost perfectly. **c** Normalised mean bubble size growth. The black solid line is a 1/3 power law, which is the lower limit for diffusion-limited coarsening. Two repeats of the experiments with *ϕ* = 0.70 are shown. The dashed line corresponds to the mean bubble growth measured for an aqueous quasi-2D foam having no oil in the continuous phase (see Supplementary Fig. 1b, c). Inset: Temporal evolution of the total number of measured bubbles, where the limit for diffusive ripening is given by the −2/3 law. Videos of the coarsening foams can be found in Supplementary Information.

Photographs of three foams at different oil fractions, taken at *t* = *t*_2D_, are shown in Fig. 2a. The pictures at *ϕ* = 0.65 and 0.75 are similar and the typical cellular foam structure can be recognised. But at *ϕ* = 0.85 the foam already has some peculiar features, such as elongated bubble shapes, which we will return to later on. Despite the morphological differences, the bubble size distributions at this point are very similar in all the foams considered. This can be seen from the distribution functions of the dimensionless radius *R*/〈*R*〉 (Fig. 2b). These are also comparable to steady-state distributions in aqueous quasi-2D foams, as shown in Supplementary Fig. 1a or in the literature^24^.

The evolution of the foams after *t* = *t*_2D_ depends on *ϕ* as observed with *ϕ* = 0.65 (Supplementary Movie 1), *ϕ* = 0.70 (Supplementary Movie 2), *ϕ* = 0.75 (Supplementary Movie 3), *ϕ* = 0.80 (Supplementary Movie 4), *ϕ* = 0.85 (Supplementary Movie 5). The temporal evolution of the average radius normalised by the average radius at *t* = *t*_2D_ of the quasi-2D foams changes considerably with *ϕ* (Fig. 2c). The aqueous foam and the foam made from the least elastic emulsion at *ϕ* = 0.65 evolve the most rapidly and with a similar apparent power law. However, the exponent is smaller than the expected *t*^1/2^ for dry foams and closer to *t*^1/3^ expected for very wet foams, although the foam is rather dry. This is a consequence of finite-size effects observed in moderately dry quasi-2D foams, where the vertical film size decreases as the foam coarsens at a constant liquid fraction^25^. Even though our systems are dry enough for adjacent bubbles to share thin films, all samples have coarsening rates equal to or below the prediction for dilute bubble dispersions. This is the lowest possible rate for diffusion-limited growth, and any change from diminished gas transport in the emulsion would only shift the curves, but not change the power laws observed. The foams made from emulsions at higher *ϕ* evolve very slowly, and with continuously slowing coarsening rates with increasing bubble size. As the liquid fraction in the foams is very similar, the differences between the samples are due to the emulsion.

**Fig. 3:**
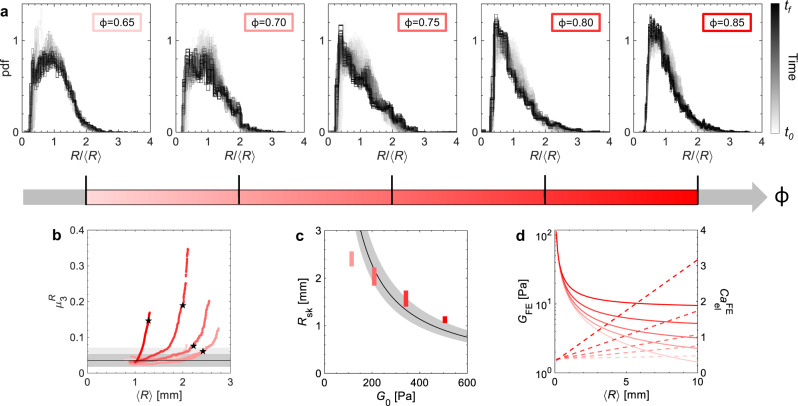
Evolution of bubble size distributions. **a** Time evolution of the dimensionless bubble size distributions in the foams made from emulsions at different *ϕ*. The curves are plotted with a grey scale proportional to the foam age, where black corresponds to the end of the image acquisition *t*_*f*_. **b** Third moment ${\mu }_{3}^{R}$ versus the mean bubble radius. The solid black line indicates the average value at early times. The grey zones show the ±50% and +100% values, which are used to estimate *R*_sk_ in (**d**). The ${\mu }_{3}^{R}$ have black stars, which indicate the values at *t* = 96 h. **c** Impact of emulsion elastic modulus on *R*_sk_. The length of the data markers indicates the impact of changing the criteria for the deviation of ${\mu }_{3}^{R}$ from its plateau. The black line shows the prediction with ${\rm {C{a}}}_{{{{{{{{\rm{el}}}}}}}}}^{{{{{{{{\rm{FE}}}}}}}}}=0.54$ and the grey region the impact of changing liquid fraction on it. **d** Evolution of the expected foam elastic modulus *G*_FE_ (solid lines) and of the foam elastocapillary number ${\rm {C{a}}}_{{{{{{{{\rm{el}}}}}}}}}^{{{{{{{{\rm{FE}}}}}}}}}$ (dashed lines) as the mean bubble size grows because of coarsening, for the different *ϕ*.

We have a high number of bubbles *N* throughout the coarsening process (inset of Fig. 2c), which allows us to measure bubble size distributions in time with good statistics. We thus compare the evolution of the dimensionless bubble size distributions in the foams made from the different emulsions (Fig. 3a). It is clear that the elasticity of the continuous phase has an impact on the distributions, which, unlike aqueous foams, do not head towards self-similar distributions obtained at steady state^24^. Although the exact evolution depends on *ϕ*, in all of them a shift of the peak to smaller *R*/〈*R*〉 can be observed. This shift implies an accumulation of smaller bubbles over time, which indicates a delay in their disappearance that is at the origin of the slow coarsening evolutions observed. The distributions cannot be fitted with a single distribution function throughout the ageing process, and we do not observe a division into the two populations in the bubble-size distributions. We choose to quantify the change in the shape of the distribution through its third central moment ${\mu }_{3}^{R}$, which is sensitive to the distribution asymmetry or skew and, in this case, to the presence of small bubbles. By plotting ${\mu }_{3}^{R}$ as a function of the average bubble radius (Fig. 3b), we can see that ${\mu }_{3}^{R}$ is initially around 0.03 for all the foams, but as the bubble size increases it departs from this plateau. Without emulsion ${\mu }_{3}^{R}$ remains constant, as shown in Supplementary Fig. 1d. The higher *ϕ*, the smaller the bubble size at which the skew deviates. We note that at *ϕ* = 0.65 a deviation is observed as the bubbles reach around 2 mm, but this is due to the onset of coalescence and hence the physical origin is different than in the other samples. We define the radius at which ${\mu }_{3}^{R}$ has increased by 50% from its average value (from 0.036 to 0.053) as *R*_sk_. This gives us a characteristic radius at which the foam evolution has deviated from that of classical foams. *R*_sk_ decreases with increased *ϕ*, and hence higher *G*_0_ (Fig. 3c).

We have quantified the number of neighbour-changing rearrangement events (T1s), which are an important feature of coarsening foam dynamics and stress relaxation. At the start of the experiment, the number of events depends on oil volume fraction (higher *ϕ* less T1s). However, before *R*_sk_ is reached the number of T1s has become so low that we do not have sufficient statistics to quantify them (despite *N* > 1000) and they have effectively stopped. This suggests that the bubbles are shrinking and expanding without much reorganisation, which is why we expect the departure from standard foam coarsening to happen once the elasticity of the continuous phase becomes large compared to Laplace pressure. However, in foam, the bubbles are not surrounded by a continuous emulsion phase of constant elastic modulus, but by other bubbles forming a foam. The elastic modulus of a 3D foam with an elastic continuous phase can be described by an additive model combining bubble *G*_F_ and continuous phase *G*_E_ contributions as *G*_FE_ = *G*_F_ + *G*_E_^15^. The foam elasticity is modelled by ${G}_{{\rm {F}}}=1.6\frac{\gamma }{\langle R\rangle }(1-\epsilon )(0.36-\epsilon )\equiv f(\epsilon )\frac{\gamma }{\langle R\rangle }$^26^, and the continuous phase by *G*_E_ = *ϵ*^2^*G*_0_^18^, where *ϵ* is the foam liquid fraction. Despite the structural differences between quasi-2D and 3D foams we use the 3D foam description to capture the dependency of *G*_FE_ on 〈*R*〉 and *G*_0_. For simplicity we do not use the coupling function introduced by Gorlier et al.^15^, as it only has a weak effect on our estimated moduli and as the function could be system dependent. The elastic moduli of foam *G*_FE_ calculated with the foam liquid fraction *ϵ* and the elastic moduli *G*_0_ corresponding to our emulsions are shown as a function of the average bubble radius in Fig. 3d. At small 〈*R*〉 the *G*_FE_ are all close as they are dominated by the elasticity of the bubbles, while at larger bubble sizes the foam elasticity depends strongly on *ϕ*.

As the foams coarsen the average bubble size increases and elastic effects from the emulsion become relatively more important. In order to compare the latter to Laplace pressure, we can construct a second elastocapillary number as ${\rm {C{a}}}_{{{{{{{{\rm{el}}}}}}}}}^{{{{{{{{\rm{FE}}}}}}}}}={G}_{{{{{{{{\rm{FE}}}}}}}}}/(\gamma /R)=f(\epsilon )+\langle R\rangle {G}_{0}{\epsilon }^{2}/\gamma$, which increases with 〈*R*〉 (Fig. 3d). It has been shown that monodisperse foams stop coarsening once ${\rm {C{a}}}_{{{{{{{{\rm{el}}}}}}}}}^{{{{{{{{\rm{FE}}}}}}}}} \, \approx \, 0.8$^9^. We can describe the evolution of *R*_sk_ with *G*_0_ assuming a critical ${\rm {C{a}}}_{{{{{{{{\rm{el}}}}}}}}}^{{{{{{{{\rm{FE}}}}}}}}}=0.54$ (Fig. 3c) unique to all *ϕ*. Therefore, in our foams, elastic effects from the continuous phase become visible in the bubble size distributions once 2*G*_FE_ ≈ *γ*/〈*R*〉. The experiment at *ϕ* = 0.65 stops at ${\rm {C{a}}}_{{{{{{{{\rm{el}}}}}}}}}^{{{{{{{{\rm{FE}}}}}}}}} \, \approx \, 0.4$, below the critical ${\rm {C{a}}}_{{{{{{{{\rm{el}}}}}}}}}^{{{{{{{{\rm{FE}}}}}}}}}$, which is why for these foams the distribution is unchanged (Fig. 3a). The foams also never stop coarsening despite reaching ${\rm {C{a}}}_{{{{{{{{\rm{el}}}}}}}}}^{{{{{{{{\rm{FE}}}}}}}}}=0.8$, however, this is because they are polydisperse.

**Fig. 4:**
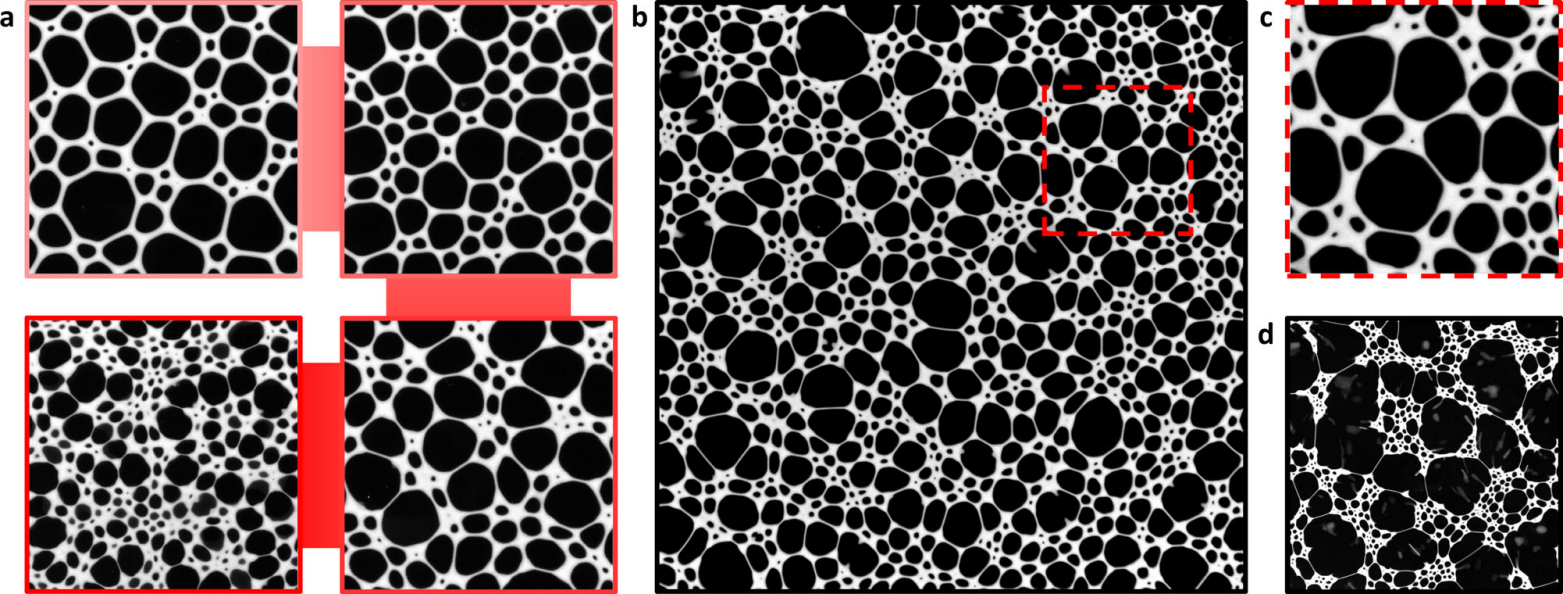
Foamed emulsion structure. **a** Sample structure after 96 h at different *ϕ*. From top left to bottom left, clockwise: *ϕ* = 0.70, 0.75, 0.80, 0.85. The edge of each frame is 4 cm. The corresponding skew of the distributions is indicated by a star in Fig. 3b. At higher *ϕ* the foam structure becomes highly unconventional with unrelaxed bubble shapes and uneven emulsion distribution. **b** Sample at *ϕ* = 0.80 after 5 days. Chains of large bubbles and regions of small bubbles become clearly visible. Edge size 12 cm. **c** Enlargement showing the emulsion bulge at the ends of the thin Plateau borders between large bubbles. Edge size 3 cm. **d** Sample having *ϕ* = 0.80 made with sunflower oil, after 12 days. The edge of the frame is 13 cm. Segregation into regions of large and small bubbles is complete.

Bubble-size polydispersity in our samples also plays a crucial role in their structural evolution. Since *G*_FE_ decreases with *R* (Fig. 3d), in a polydisperse foam larger bubbles are easier to deform than smaller ones. This spatial heterogeneity in the foam’s mechanical properties affects the coarsening process causing a peculiar evolution of the foam structure. Bubbles will grow preferentially towards weaker regions, thus towards other large bubbles. The resulting heterogeneous ageing, which leads to segregation between smaller and larger bubbles, becomes visible after 96 h of ageing (Fig. 4a) for samples with *ϕ* ≥ 0.75. The clusters of small bubbles enclosed by the chains of larger ones are striking at *ϕ* = 0.80 (Fig. 4b).

The emulsion elasticity also changes the structure more locally. We observe the emulsion bulging into the bubbles at the vertices (Fig. 4c). Such inversion of curvature is another sign of the easier deformability of the bubbles instead of the emulsion, and departure from capillary-mediated Plateau’s laws. We can also observe that the liquid is unevenly distributed in the foams, as the smaller bubbles are surrounded by more emulsion than the large ones. In the foams made from high *ϕ* emulsions, this will increase the local elastic modulus further and accentuate the bimodal coarsening.

The inhomogeneous evolution of the foam due to the heterogeneity in mechanical properties with bubble size resembles the elastic ripening recently observed by Rosowski et al. at a microscopic scale^27^. In their work ripening is arrested in elastic regions, and continues in the less elastic ones, where the inhomogeneity is created by changing the polymer matrix while in our case the inhomogeneity is inherently due to the variation in the response of the foam with bubble size.

If the foams are left to evolve for a very long time, the larger bubbles will keep growing until they start to coalesce, leaving the islands of small bubbles behind (Fig. 4d). The photograph is reminiscent of structures observed during the final stages of viscoelastic phase separation identified by Tanaka^28^ in systems with high dynamic asymmetry. He proposed its occurrence in foams despite the difference in the coarsening processes from the polymer phase separation or colloidal gelation previously studied, and the images could be a first indication of such a process at millimetric length scales.

We show that the viscoelasticity of the foam’s continuous phase leads to a slowing down of coarsening and a radical change in the foam structure. Once a critical ${\rm {C{a}}}_{{{{{{{{\rm{el}}}}}}}}}^{{{{{{{{\rm{FE}}}}}}}}}$ is reached, the bubble size distributions show the accumulation of smaller bubbles, which eventually segregate from the larger ones. This is caused by the interplay between bubble size and foam elasticity, which combined with bubble size polydispersity results in highly heterogeneous materials. Stress buildup in the unrelaxed structures will be transferred to the foams if dried or solidified resulting in mechanical weakness. The deviation from the traditional cellular foam structure can have other implications on the material properties, so understanding and control of the structural evolution are crucial for the safe design of foamy materials.

## Methods

### Materials

Air bubbles and oil droplets (rapeseed oil from Brassica Rapa, Sigma-Aldrich) are dispersed in an aqueous solution containing 30 g/L of sodium dodecyl sulfate (SDS, from Sigma-Aldrich).

### Emulsion generation

Concentrated O/W emulsions are generated by mechanically mixing the oil and the aqueous surfactant solution with the double syringe method. This technique allows to control the oil volume fraction and ensures good reproducibility of the samples. The resulting droplet size distribution is measured with laser diffraction with a Mastersizer 3000E (Malvern Panalytical) equipped with a Hydro SM wet dispersion unit. The size distribution is polydisperse, with a span, defined as *d*(90%)−*d*(10%)/*d*(50%), ranging from 1.2 to 1. The surface-weighted mean drop diameter ranges between 3 and 5 μm. Both the average size and the distribution width decrease with increasing *ϕ*, consistently with emulsification in turbulent flow^29^. We measured the surface tension of the air/emulsion interfaces using a pendant drop tensiometer (Tracker, Teclis, France). The surface tension of the emulsions is systematically lower at 30 mN/m than that of the SDS solution/air surfaces, which are at 34 mN/m suggesting the presence of a mixed SDS-oil surface layer. We note that this is not indicative of a particle stabilisation of the interfaces by intact oil drops, and once the emulsion drops enter the gas/liquid surface they will break up and spread.

### Emulsion rheology

The mechanical properties of concentrated emulsions are measured with an MCR302 rheometer (Anton Paar). The emulsion storage and loss moduli are obtained by performing oscillatory strain sweep tests (frequency 1 Hz, amplitude from 10^−5^ to 1) in a cylindrical Couette geometry (CC27), with a gap of 1.1 mm. All measurements are performed at (20.3 ± 0.1) °C. A more complete rheological characterisation is shown in Supplementary Fig. 2a–e.

### Foam generation

Emulsions are foamed with a planetary kitchen mixer (Kenwood MultiOne 1000 W). The mixing speed is gradually increased from the minimum to the maximum level, and after 25 min of whipping, up to roughly 90% of air is incorporated inside the emulsion, without affecting the droplet size distribution. The final foam liquid fraction is measured by weight: a glass container of known volume *V*_foam_ is filled with freshly made foamed emulsion and then weighed, so that, if the gas density is assumed to be zero, the mass of the foam liquid content *m*_em_ is measured. We assume the emulsion density to be given by the weighted sum of the density of its two components, namely *ρ*_em_ ≃ *ϕρ*_oil_ + (1−*ϕ*)*ρ*_water_, so that the foam liquid fraction is retrieved through the relation *ϵ* = *m*_em_/(*ρ*_em_*V*_foam_). The final foam liquid fraction ranges between 9% and 13%, and slightly increases with *ϕ*, consistently with the increase of emulsion bulk viscosity^30^.

### Imaging and image treatment

Freshly generated foamed emulsions are carefully sandwiched between two square glass plates (edge length 24 cm), separated by a rubber joint of thickness 1 mm which sets the cell gap, d_gap_. The cell is then placed between two square metal frames which are screwed to keep the cell closed. The cell is then put under a digital camera (Basler acA3800—14 μm, resolution 3840 × 2748 pixel) equipped with a lens (TAMRON 16 mm *f*/1.4), and a square of LED lights provides a rather uniform illumination from above. A photo of the foamed emulsion is taken every 3 min at the early stage, and then every 30 min at the late coarsening stage.

Custom-made MATLAB scripts are used for processing the images. A first image pre-treatment is carried out by cropping the raw frames around a region of interest, adjusting the contrast, and obtaining the 2D foam skeleton through a watershed algorithm. The skeletonised frames are then processed with a second MATLAB script based on the built-in function *regionprops* to retrieve the area of each gas cell, from which the equivalent radius is calculated as $R=\sqrt{A/\pi }$.

## Supplementary information

Supplementary Information

Description of Additional Supplementary Files

Supplementary Movie 1

Supplementary Movie 2

Supplementary Movie 3

Supplementary Movie 4

Supplementary Movie 5

## Data Availability

The videos of the coarsening foams used in this study have been deposited in the Zenodo database using the following link 10.5281/zenodo.7535841. The rheological data generated in this study are provided in the Supplementary Information file.
