# Supplementary material for: Viscoelastic coarsening of quasi-2D foam
Source: Nat Commun. 2023 Feb 28;14:1125. doi: 10.1038/s41467-023-36763-y (PMC9975196; doi:10.1038/s41467-023-36763-y)
Supplement: Supplementary file 1 — Supplementary Information [file 41467_2023_36763_MOESM1_ESM.pdf]

Supplementary Information

January 30, 2023

Manuscript NCOMMS-22-27980-T
Title: Viscoelastic coarsening of quasi-2D foam.
Nature Communications

1 Quasi-2D aqueous foam coarsening

The coarsening of an aqueous quasi-2D foam without emulsion was characterised to provide a comparison for the behaviour of the foamed emulsions. The foam was made from an SDS solution at 30 g/L following the experimental procedure for the emulsion foams. The liquid fraction in the foam is comparable to the foamed emulsion samples at around 11%. The evolution of the bubble size distribution, number of bubbles, average radius and skewness of the bubble size distribution are shown in Figure 1.

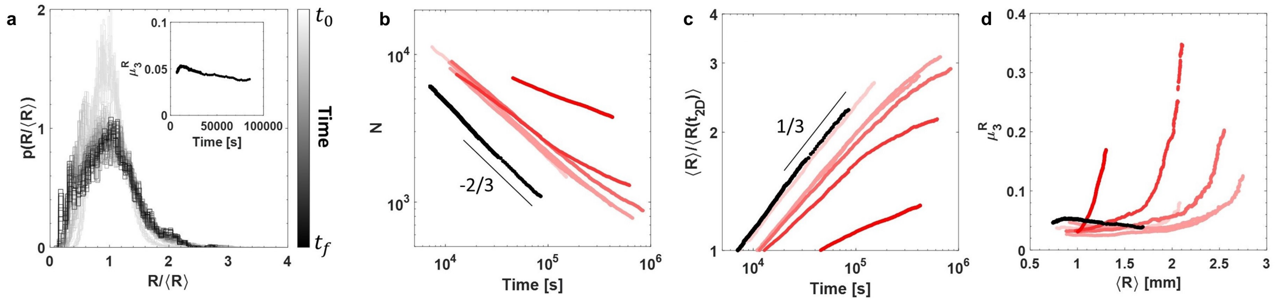

Figure 1: **Comparison with quasi-2D aqueous foam.** **a**. The bubble size distribution of the aqueous foam in time. In the inset the temporal evolution of μ_3^R. **b**. The evolution of the number of bubbles. **c**. Normalised bubble size as a function of time. **d**. Evolution of skew of the bubble size distribution. In figures **b-d** the black data is for the aqueous foam and the red data sets are for the foamed emulsions at $\phi = 0.65$ to 0.85 with a darker red indicating an increasing ϕ.

The bubble size distribution evolves at early times, however reaches a stationary state as shown by the darkest lines in Figure 1a. The shape of the distribution is in agreement with the one found for quasi-2D foams in their scaling state [1, 2]. The inset shows that the skewness of the distribution remains almost constant throughout the experiment. The evolution of the number of bubbles and the average bubble radius are coherent with an evolution of the radius as $t^{1/3}$. In Figure 1d the third moment μ_3^R of the aqueous foam deceases weakly with R, and it is very different from the rise in skewness exhibited by the foamed emulsions at later stages of their evolution.

2 Emulsion rheology

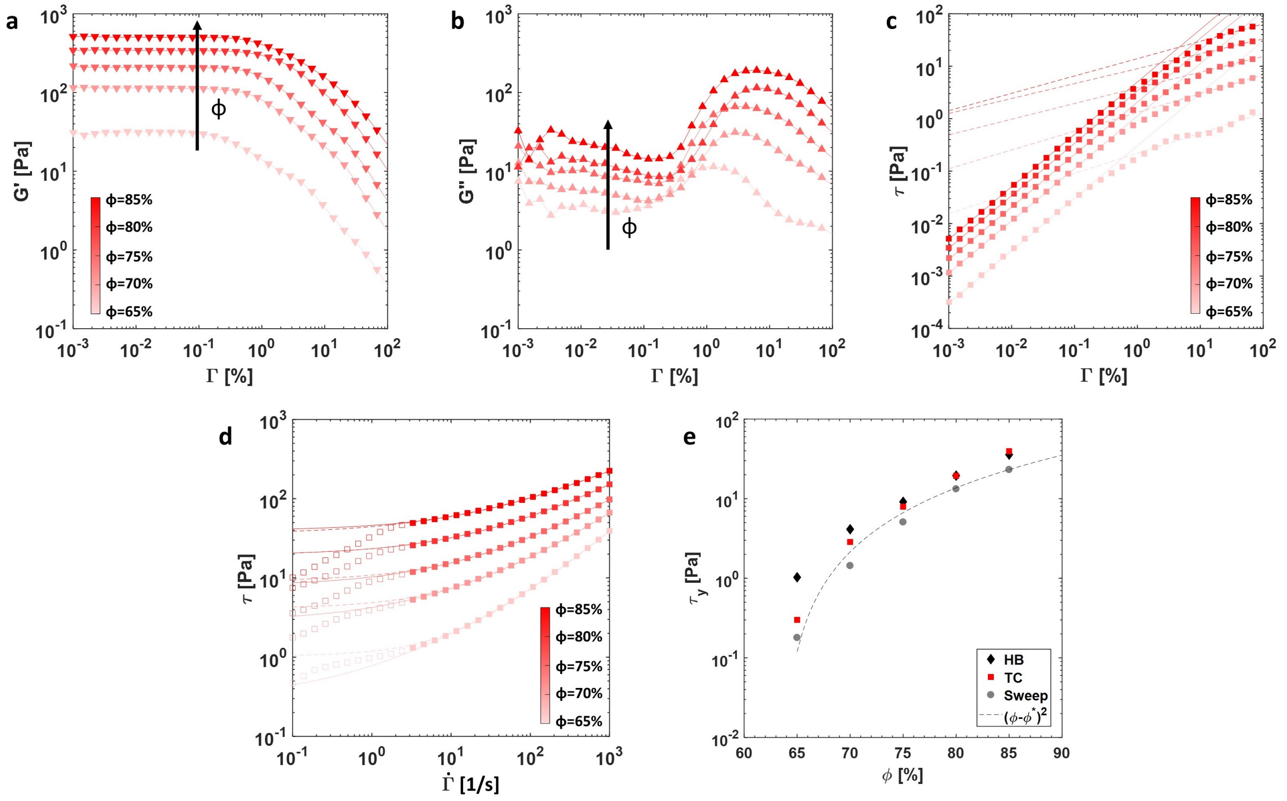

Figure 2: **Emulsion rheology.** **a-c**, Amplitude sweep results. **a**, G' and **b**, G'' versus strain Γ. **c**, Shear stress τ versus strain Γ. The yield stress τ_y can be obtained by the intersection of the two lines corresponding to the linear fit in the linear viscoelastic regime (solid lines) and above yielding (dashed lines). **d**, Emulsion flow curves: shear stress τ vs shear rate $\dot{\Gamma}$ at different emulsion oil fractions ϕ. Experimental data are fitted with both Herschel-Bulkley (HB) model (dashed lines) and three-component (TC) equation (solid lines). The solid symbols indicate the fitted data range, while the open square data points are excluded because of emulsion slip. **e**, Emulsion yield stress. The graph shows the values of τ_y calculated from both the sweep tests and the flow curves. The data agree well with the predicted scaling (dashed line) [3].

Amplitude sweep tests were performed in a cylindrical Couette geometry, at a constant frequency equal to 1 Hz. Results are shown in Figure 2 (a,b) where we plot the storage and loss moduli, G' and G'', measured for different strain amplitudes. The moduli are fixed mainly by the oil fraction and the size of the emulsion drops. For a given oil fraction the emulsion generation method ensures a good reproducibility of the drop size distribution. This has been experimentally verified. Therfore the variation in G' and G" are expected to be below 10%. This means that in Figure 1a of the main article the symbol size encompasses the error bars. A first rough estimate of the emulsion yield stress can be obtained from the amplitude sweep test data [4]. In Figure 2c we plot the measured shear stress τ versus the amplitude of the applied shear strain Γ. By fitting the shear stress curve both in the linear viscoelastic regime and in the part of the curve above yielding, we can get the yield stress τ_y^{sw} as the ordinate of the intersection between the two lines.

However, for a better estimate of the yield stress, we also measured the flow curve of our emulsions. By using the same geometry, a constant shear rate is applied for 10 seconds at 30 logarithmically spaced values starting from 1000 s^{-1} to 0.1 s^{-1} and we measure the resulting shear stress. Results are shown in Figure 2d. To obtain the yield stress τ_y, one

ϕ [%]	G' [Pa]	G'' [Pa]	τ_y^{sw} [Pa]	τ_y^{HB} [Pa]	τ_y^{TC} [Pa]
65	31	4	0.2	1	0.4
70	114	7	1.4	4.1	2.9
75	209	11	5.1	9.1	7.9
80	342	14	13.3	19.5	19.4
85	506	24	23.2	35.8	39.5

Table 1: **Emulsion rheological properties.** Values of the storage and loss moduli in the linear viscoelastic regime, and the yield stress obtained with different methods, for each emulsion oil fraction and oil type.

can traditionally fit the flow curve with the empirical Herschel–Bulkley (HB) equation $\tau = \tau_y^{\text{HB}} + K\dot{\Gamma}^n$ or with a recently proposed three component (TC) model described by the equation $\tau = \tau_y^{\text{TC}} + \tau_y^{\text{TC}}(\dot{\Gamma}/\dot{\Gamma}_c) + \eta_{bg}\dot{\Gamma}$ [5]. The flow curves of our emulsions show a decay with decreasing shear rate $\dot{\Gamma}$ for $\dot{\Gamma} < 1$ s^{-1} which could be due to emulsion slip, as pointed out also in ref. [5]. These data points are thereby not considered in the fit. We fit the reliable data range with both models and the resulting yield stress values are summarised in Table 1, together with the values calculated from the amplitude sweeps.

The ϕ dependency of the emulsion yield stress is shown in Figure 2e, where we can see that data are globally in good agreement with the predicted scaling $\tau_y \sim (\phi - \phi^*)^2$ [3].

References

[1] Glazier, J. A., Anderson, M. P. & Grest, G. S. Coarsening in the two-dimensional soap froth and the large- Q potts model: A detailed comparison. *Philosophical Magazine B: Physics of Condensed Matter; Statistical Mechanics, Electronic, Optical and Magnetic Properties* **62**, 615–647 (1990).

[2] Roth, A. E., Chen, B. G. & Durian, D. J. Structure and coarsening at the surface of a dry three-dimensional aqueous foam. *Phys. Rev. E* **88**, 062302 (2013).

[3] Mason, T., Bibette, J. & Weitz, D. Yielding and flow of monodisperse emulsions. *Journal of Colloid and Interface Science* **179**, 439–448 (1996).

[4] Dinkgreve, M., Paredes, J., Denn, M. M. & Bonn, D. On different ways of measuring "the" yield stress. *Journal of Non-Newtonian Fluid Mechanics* **238**, 233–241 (2016). Viscoplastic Fluids From Theory to Application 2015 (VPF6).

[5] Caggioni, M., Trappe, V. & Spicer, P. T. Variations of the Herschel–Bulkley exponent reflecting contributions of the viscous continuous phase to the shear rate-dependent stress of soft glassy materials. *Journal of Rheology* **64**, 413–422 (2020).
