# Supplementary material for: Viscoelastic coarsening of quasi-2D foam
Source: Nat Commun. 2023 Feb 28;14:1125. doi: 10.1038/s41467-023-36763-y (PMC9975196; doi:10.1038/s41467-023-36763-y)
Supplement: Supplementary file 2 — Description of Additional Supplementary Files [file 41467_2023_36763_MOESM2_ESM.pdf]

Description of additional supplementary files:

Movies of the first 36 hours of coarsening of the foams with oil volume fractions φ = 0.65, 0.7, 0.75, 0.8 and 0.85 are shown. All movies have a time interval of 1800 seconds between consecutive frames, and they all start at $t = t_{2D}$ of the respective sample. The size of the frame edge is 15 cm for all of them.

Supplementary Movie 1. Video of the first 36 hours of foam coarsening with φ = 0.65 starting at $t = t_{2D}$. The size of the frame edge is 15 cm.

Supplementary Movie 2. Video of the first 36 hours of foam coarsening with φ = 0.7 starting at $t = t_{2D}$. The size of the frame edge is 15 cm.

Supplementary Movie 3. Video of the first 36 hours of foam coarsening with φ = 0.75 starting at $t = t_{2D}$. The size of the frame edge is 15 cm.

Supplementary Movie 4. Video of the first 36 hours of foam coarsening with φ = 0.8 starting at $t = t_{2D}$. The size of the frame edge is 15 cm.

Supplementary Movie 5. Video of the first 36 hours of foam coarsening with φ = 0.85 starting at $t = t_{2D}$. The size of the frame edge is 15 cm.
